# Return to Work One Year after Moderate to Severe Traumatic Injury in a Working Age Population

**DOI:** 10.3390/jcm13175308

**Published:** 2024-09-07

**Authors:** Christoph Schäfer, Håkon Øgreid Moksnes, Mari Storli Rasmussen, Torgeir Hellstrøm, Cathrine Brunborg, Helene Lundgaard Soberg, Olav Røise, Cecilie Røe, Nada Andelic, Audny Anke

**Affiliations:** 1 Department of Physical Medicine and Rehabilitation, Oslo University Hospital, 0424 Oslo, Norway; hakmok@ous-hf.no (H.Ø.M.); masras@ous-hf.no (M.S.R.); uxhetz@ous-hf.no (T.H.); helus@oslomet.no (H.L.S.); cecilie.roe@medisin.uio.no (C.R.); nadand@ous-hf.no (N.A.); 2 Faculty of Health Sciences, Department of Clinical Medicine, UiT The Arctic University of Norway, Postboks 6050 Langnes, 9037 Tromsø, Norway; audny.anke@uit.no; 3 Department of Physical Medicine and Rehabilitation, University Hospital of North Norway, Postboks 100, 9038 Tromsø, Norway; 4 Institute of Health and Society, Research Centre for Habilitation and Rehabilitation Models & Services (CHARM), Faculty of Medicine, University of Oslo, 0373 Oslo, Norway; 5 Faculty of Health Sciences, Oslo Metropolitan University, 0130 Oslo, Norway; 6 Oslo Centre for Biostatistics and Epidemiology, Research Support Services, Oslo University Hospital, 0424 Oslo, Norway; uxbruc@ous-hf.no; 7 Norwegian Trauma Registry, Division of Orthopaedic Surgery, Oslo University Hospital, 0424 Oslo, Norway; olav.roise@medisin.uio.no; 8 Institute of Clinical Medicine, Faculty of Medicine, University of Oslo, 0130 Oslo, Norway

**Keywords:** rehabilitation, traumatic injuries, return to work, function, cohort study

## Abstract

**Background/Objectives:** Physical trauma may cause long-term disabilities. The importance of place of residence in the return to work after injuries is little researched. The primary aims of this study were to describe return to work or school (RTW) at 6 and 12 months after moderate to severe traumatic injury and to investigate demographic and injury-related predictors for RTW with an initial focus on geographic centrality of residency. The secondary aim was to investigate the association between RTW and functioning. **Methods:** A prospective cohort study conducted at two Norwegian trauma centres. Inclusion criteria: age 18 to 70 years, at least a two-day hospital stay and a New Injury Severity Score > 9. Information about centrality, demographics, injuries, and return to work were collected. Associations between possible predictors and RTW were assessed using binary logistic regression. **Results:** Of the 223 participants, 68% had returned to work after 6 months and 77% after 12 months. Twelve-month RTW was 89% after thorax/abdomen injuries, 78% after extremity/spine injuries and 73% after head injuries. More central residency was a significant predictor for RTW in univariable but only within the extremity/spine injury subgroup in multivariable analysis. Negative factors were age, having a blue-collar job, number of injuries and rehabilitation complexity. Function 12 months post-injury was associated with RTW in the multivariable model. **Conclusions:** RTW after one year was high in all major trauma groups. Demographic and injury-related factors were more important predictors of RTW than centrality of residency. Blue-collar workers and patients with multiple injuries and high rehabilitation complexity should be given special attention to support RTW.

## 1. Introduction

Patients who have suffered moderate to severe traumatic injuries experience long-term limitations in physical function, participation and mental health [1]. Participation in work is one of the key outcomes both at the individual and societal level [2,3]. The UN Convention On The Rights of Persons With Disabilities demands promotion of vocational and professional rehabilitation as well as return-to-work programmes for persons with disabilities [4]. Various sociodemographic aspects and the type of injury influence return to work (RTW) after one year both in populations with mainly mild injuries and populations with more severe injuries [5,6,7,8,9]. In previous studies, RTW after 12 months has been reported between 66% (median Injury Severity Score (ISS) of 17) [6,10], 74% (mean ISS of 28) [11], 76% (median ISS of 5) [5] and 80% (mean ISS of 25) [7]. RTW is only partly dependent on injury severity and grade of disability [7] and the type of injury is a variable determinant for RTW outcomes. While patients living in rural areas have a higher in-hospital mortality rate following trauma than patients living in urban areas [12], long distances may reduce participation in follow-up treatment [13]. There is limited knowledge of how centrality/geographic place of residence impacts disability and RTW after injuries [12,14]. A study based on insurance databases for compensation claims after accidents at work reported faster RTW for patients who had suffered fractures at work and lived in rural areas in the USA [15]. Another study from the USA showed that patients with work-related fractures who received physical medicine and rehabilitation treatment returned to work faster when living in rural areas [16]. In contrast, other studies from the USA [17] and Canada [18,19], also based on insurance databases for workers’ compensation claims, showed that employees with work-related injuries had the lowest number of compensated disability days when living in metropolitan areas. A study from Australia [20] found no differences in RTW after 18 months for patients who had rehabilitation for traumatic brain injury in relation to urban or rural places of residence. To our knowledge, no studies have investigated geographic centrality as a predictor for RTW in a general trauma centre population with moderate and severe injuries.

Several studies have investigated the role of injury type as a predictor for RTW following trauma. De Munter et al. [5] found tibia and complex foot or femur fractures, as well as stable vertebral fractures/disc injuries, to be negative predictors for RTW among trauma patients in the Netherlands. In Australia, Collie et al. [6] identified head and spinal cord injuries as predictive factors for RTW, based on data from the Victorian State Trauma Registry. However, Holtslag et al. [7] discovered that while spinal cord injuries were the strongest determinant for RTW at hospital discharge, injury types were not predictive during follow-up 12 to 18 months post-injury among severely injured trauma survivors. Dinh et al. [8] found that upper limb injuries were associated with reduced odds of RTW at 3- and 6-months post-injury, while Uleberg et al. [9] reported that patients with severe head trauma had a longer median time for RTW compared to those without. Finally, Gabbe et al. [10] observed that spinal cord-injured patients had the lowest proportion of RTW over time, while patients with chest and/or abdominal injuries had the highest. Although the choice of predictors analysed and predictors found significantly varies between studies, these findings highlight the importance of considering injury type when assessing RTW outcomes. 

Personal factors that have been shown to positively predict RTW after traumatic injury are high educational level, male gender and absence of comorbidity [5]. On the contrary, older age, female gender, receipt of compensation, greater socioeconomic disadvantage, low-prestige occupation, blue-collar jobs and presence of comorbidity were negative predictors [6,7,10,21]. The importance of disability/function after traumatic injury for RTW is not completely clear [7,22].

The primary aims of this study of a trauma centre cohort with moderate to severe traumatic injuries were to

- describe full or partial RTW at 6 and 12 months after traumatic injury.
- investigate demographic and early injury-related predictors for RTW at 12 months with an initial focus on the centrality of the living area, both overall and for specific types of injury based on the most severe injury.

The secondary aim was to investigate the association between 12 months RTW and functioning. 

## 2. Materials and Methods

### 2.1. Design and Setting

A prospective, observational cohort study was conducted at two trauma centres in Norway: Oslo University Hospital (OUH) and the University Hospital of North Norway (UNN), receiving patients in the southeast and northern regions of the country, respectively. The protocol article for the main study has been previously published [23]. The study was approved by the Committee for Medical and Health Research Ethics, Health Region South East (reference number 31676 https://rekportalen.no/#prosjektbibliotek/prosjektregister, accessed on 14 August 2024) and the Institutional Data Protection Officers at OUH and UNN (approval numbers 19/26515 and 02423). This study was funded by the South-Eastern Norway Regional Health Authority, Helse Sør-Øst RHF, Grant no. 2019043. The STROBE checklist was used to report this study [24].

### 2.2. Participants

This study included 243 patients aged 18 years to 70 years, residents in Norway and in remunerative work or education at the time of injury, admitted directly to the regional trauma centres or transferred from local hospitals within 72 h after injury, with at least a two-day hospital stay at the trauma centre and a New Injury Severity Score (NISS) > 9 [25]. The NISS criteria pertain to patients with moderate to severe injuries. The 1-year inclusion period spanned from 1.1.20 to 31.12.20 at OUH and from 1.2.20 to 31.1.21 at UNN. Exclusion criteria were age below 18 years or above 70 years, being not in work or education, non-residents of Norway, insufficient command of Norwegian/English language and death during the stay in the acute departments of the trauma centre or before follow-up. Patients or their relatives, if patients were unable to consent by themselves, were informed about the study orally and in writing and asked for written consent.

### 2.3. Data Collection

Patients were identified via the project’s team via participation in trauma department meetings for reporting and planning action related to trauma patients, lists of newly hospitalised patients in the trauma department and admission diagnoses of trauma in the medical records system. Most identified patients had been processed by the trauma team on arrival at the hospital. Patients who fulfilled the inclusion criteria were assessed.

### 2.4. Demographics, Comorbidity and Injury-Related Variables

Demographic variables were collected from the patient’s electronic records, including age, gender, marital status (living alone or with someone), educational level, occupation and job type (blue-collar/white-collar) Blue-collar jobs were defined as those with high physical demands, for example, craftsmanship, productive industries and care-giving jobs. Being a student was considered a white-collar job [26]. Any missing data about participant characteristics were obtained in the 6-month follow-up interviews. Pre-injury health was estimated with the Norwegian version of the American Society of Anaesthesiologists Physical Status Classification System (ASA) as described in the definition catalogue for the Norwegian Trauma Registry (NTR) [27]. Pre-injury ASA was dichotomised into healthy/without substantial functional limitations (score I-II) and moderate disease/disability to severe disease/disability (score III-V) [28]. Information about RTW was collected at 6- and 12-month post-injury interviews with the help of a customised questionnaire.

### 2.5. Injury Severity

The study physicians, certified Abbreviated Injury Scale (AIS) registrars, estimated AIS scores and calculated NISS daily from Monday (including patients admitted to the trauma centre at the weekend) to Friday for eligibility for study inclusion. The NISS is a summary measure of anatomic injuries and is defined as the sum of the squares of the AIS scores of each patient’s three most severe injuries, regardless of the body region in which they occur [25]. For this study, we classified a NISS of 10–15 as moderate, 16–24 as severe and ≥25 as profound traumatic injury, as defined in the protocol article [23]. In Norway, the 2008 update of the 2005 version of the AIS is used in the trauma register [29]. The AIS codes the body region affected with injury type details and the severity of each injury. Injury severity is graded 1–6: 1 is a minor and 6 is a maximal injury. Injuries graded 1–2 are classified as minor to moderate and injuries graded 3–6 as serious to maximal. AIS scores received from the local trauma registers at OUH and UNN, 2 of 38 hospitals constituting the NTR, Ref. [30] were used for analysis. The register was recently validated with good results [31]. The most severely injured body region was identified according to the methods of Anke et al. [21] a classification based on the highest AIS score thought to be meaningful relative to disability; scores for the external body region (skin) were therefore excluded. For simplicity, injuries in the thorax and the abdomen region were classed as one group. Although not represented as a major AIS body region, injuries to the spinal cord identified by ICD-10 codes were defined as a separate group. When two or more body regions had identical AIS scores, the head was given precedence over extremity, extremity over face and face over thorax/abdomen. In contrast with Anke et al. [21], injuries to the upper and lower extremities or the spine without cord injury were collectively categorised as orthopaedic. Thus, we categorised the most severe injury to each patient as either head, orthopaedic, spinal cord, face or thorax/abdomen.

### 2.6. Assessments

#### 2.6.1. Centrality

We used the Norwegian Centrality Index NCI to determine centrality as a variable. The NCI was developed by Statistics Norway as a measure of how centrally municipalities are located, based on services and workplaces that are accessible by car to a resident within 90 min [32]. The NCI ranges from 1 to 6, where indices 1 and 2 denote the most central areas (Oslo and the other big cities in Norway) and indices 5 and 6 denote the least central areas (small rural municipalities, e.g., Nore og Uvdal or Balsfjord). The NCI category was determined based on the patient’s municipality of residence. For analysis, the six categories were collapsed into two groups: 1 and 2 (most central, referred to as NCI 1–2) and 3 to 6 (less central, referred to as NCI 3–6) [33].

#### 2.6.2. Rehabilitation Needs

The Rehabilitation Complexity Scale-Extended Trauma (RCS-E Trauma) estimates the complexity of patients’ rehabilitation needs and interventions indicated for specialised inpatient rehabilitation [34]. The scale takes into account basic care, specialist nursing, therapy, equipment needs and medical interventions. It can also be used to assess received rehabilitation services. The RCS-E Trauma reflects the seven following domains: medical needs (M, 0–6), basic care and support needs (C, 0–4) risk (cognitive or behavioural needs) (R, 0–4), skilled nursing needs (N, 0–4), number of different therapy disciplines required (TD, 0–4), therapy intensity (TI, 0–4) and equipment needs (E, 0–3). The total score is computed as the sum of item scores, with only the higher value between C and R scores used, providing a sum score range of 0–25 [34]. The RCS-E is established as a feasible and useful tool for the assessment of rehabilitation complexity in acute trauma care [35] and primary rehabilitation needs in patients with acquired brain injury [36].

RCS-E Trauma scores were estimated by physical medicine and rehabilitation specialists in accordance with clinical judgment at discharge from the acute department. The inter-rater reliability between the three rehabilitation specialists who did the scorings was calculated with intra-class correlation coefficients (ICC). Inter-rater reliability was excellent for consistency (ICC 0.933) and good for absolute agreement (ICC 0.899). The calculation was based on a random sample of 11 patients.

#### 2.6.3. Function

World Health Organisation Disability Assessment Schedule (WHODAS 2.0 12-item) scores were obtained as part of the 12-month telephone-based follow-up interview. Scores were estimated based on information given by patients or/and relatives in the interviews. The WHODAS 2.0 12-item is a generic assessment instrument that measures disability at a population level or in clinical practise. It measures the level of functioning in six domains (cognition, mobility, self-care, getting along, life activities and participation) with two items per domain. Each item is scored from 0 to 4, where 0 means no difficulty, 1 mild, 2 moderate, 3 severe and 4 indicates extreme difficulty or complete inability to perform the specified activity. The total score is the sum of the sub-scores and ranges from 0 to 48, with lower scores indicating better functioning. Total scores of 1–4 indicate mild disability, 5–9 moderate disability and 10–48 severe disability [37,38]. Scores from participants who had one item missing were imputed with the mean of the other 11 items according to the WHODAS manual. Participants who had more than 1 item unanswered were excluded. Item 12 in the WHODAS 2.0 directly evaluates the ability to work and so would interfere with the outcome RTW. We therefore removed this item. Out of a total score range between 0 and 44, we calculated the mean and SD of the remaining items for use in the analysis. Scores with one of eleven items missing were imputed with the mean score of available items.

##### Statistical Analysis

IBM SPSS Statistics Version 29 (IBM Corp., Armonk, NY, USA) was used for statistical analysis. Descriptive data are presented as proportions (percentages), medians with range respective interquartile range (IQR) and means with standard deviation (SD). The significance of differences between groups was tested with either a *t*-test, chi-squared test or Fisher’s exact test as appropriate.

Binary logistic regression analyses were performed to investigate the relationship between centrality and RTW and to adjust for possible confounding effects of demographic and injury-related variables. Univariable and multivariable binary logistic regression analyses were used to investigate possible prognostic factors associated with RTW. Analyses were performed in the total study population and within subgroups of injury type.

The following sociodemographic and injury-related factors were included in the univariable analysis: centrality, age, gender, living situation (living alone/not living alone), type of work (blue/white collar), educational level (higher education > 13 years/lower education ≤ 13 years), pre-injury ASA (dichotomized into score 1–2 vs. 3–4), substance use at the time of injury (yes/no), NISS and total number of injuries; Further the most common injured organ regions (head, extremities and spine without spinal cord damage and thorax/abdomen) with highest AIS cores. Because of the limited sample size, especially for the organ area subgroups we chose the variables with the highest assumed importance for RTW based on literature and clinical experience. In the thorax/abdomen subgroup, where only seven participants did not return to work, only the univariable analyses are presented. An additional multivariable analysis was performed, investigating whether functioning (WHODAS 11-item sum score) was significantly associated with RTW after controlling for all the other factors. The results are given as odds ratios (ORs) with a 95% confidence interval (95% CI). The level of significance was set at *p* ≤ 0.05. The degree of multicollinearity was checked using the correlation matrix and Spearman’s correlation coefficient ≥ 0.7 as a cut-off. Model fit was assessed with the Hosmer and Lemeshow goodness-of-fit test, and the degree of pseudo-explained variance was reported according to Nagelkerke R^2^.

## 3. Results

**Figure 1:**
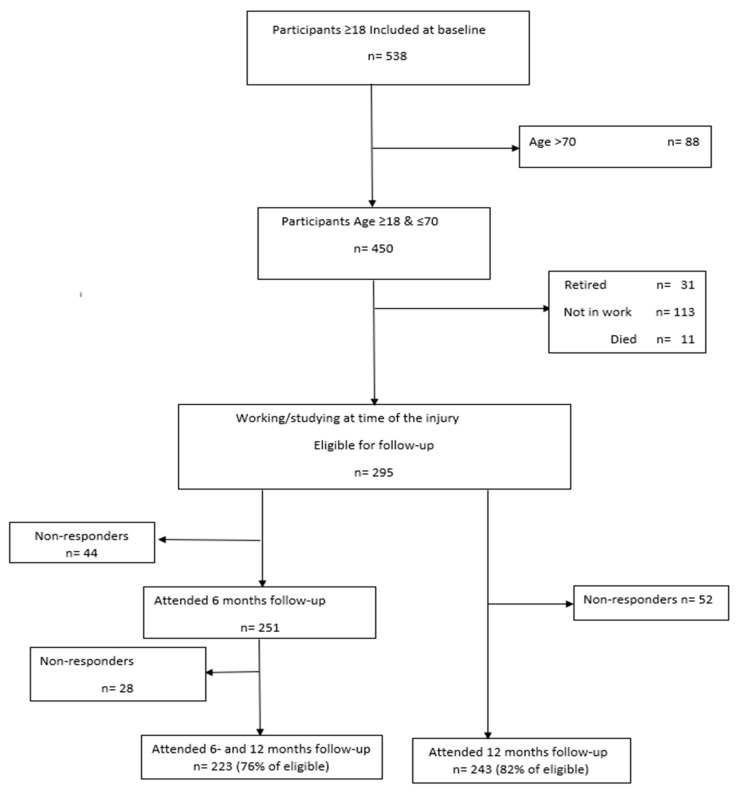
Inclusion flowchart.

Of 450 participants between 18 and 70 years, 295 were studying or at work at the time of the injury. Of these, 243 (82%) attended the 12-month follow-up and were included in the analysis (see Figure 1).

The 52 non-responders were significantly younger (mean 39 vs. 45 years, *p* = 0.003), were more likely to live alone (50 vs. 33%, *p* = 0.019), had lower education (65 vs. 45%, with education ≤ 13 years, *p* = 0.008) and higher pre-morbidity (10 vs. 3%, *p* = 0.025 with ASA ≥ 3) than the 243 participants. There were no significant differences in injury-related variables.

As shown in Table 1, the average age of the 243 participants was 45 (SD 15) years, 21% were women and 62% lived in central areas. In total, 9% were students and 42% had blue collar jobs. Injury-related characteristics of the patients are shown in Table 2. The median NISS was 22 (range 10–75), and 76% of the participants had severe injuries. The most common most severe injury was head injury, and the median total number of injuries was 5 (range 1–23).

### 3.1. Return to Work or School at 6 and 12 Months after Traumatic Injury

Of 295 eligible participants, 223 attended both the 6- and 12-month follow-up. After 6 months, 68% of the participants had returned to work with 41% in a full-time job. Out of the 187 (77%) participants who had returned to work after 12 months, 151 (62%) worked full-time and 33 (14%) worked part-time (See Table 1). Table 3 displays the RTW rates for participants in the main injury groups, who attended both follow-ups. Participants with dominant thorax/abdomen injuries had the highest overall RTW rate after 6 months (88%), with 22% of these only working part-time. After 12 months, the total RTW rate for this group was 89%, with 11% in part-time work. Participants with spinal cord injuries had an RTW rate of 79% (50% part-time) after 6 months, which increased to 85% (14% part-time) after 12 months. Participants with dominant head injuries had the lowest RTW rate, both after 6 months (59%, 28% working part-time) and 12 months (73%, 20% working part-time). Participants with dominant injuries in the extremities/spine region had RTW rates between those of the other groups: 69% after 6 months (26% working part-time) and 78% after 12 months (17% working part-time).

### 3.2. Centrality as an Early Predictor for RTW at 12 Months Post Injury

As seen in Table 4, participants living in less central areas returned to work less often than those living central (66 vs. 84%, *p* = 0.001), had lower education (proportion with education ≤13 years 63 vs. 34%, *p* < 0.001) and more often blue-collar jobs (59 vs. 32%, *p* < 0.001). They also had a higher mean NISS (28 vs. 23 *p* = 0.002) and more injuries (mean 7 vs. 6, *p* = 0.002). Estimated rehabilitation needs were higher (mean sum RCS-E Trauma at baseline, 10 vs. 7, *p* < 0.001), compared to those living centrally.

### 3.3. Other Demographic and Injury-Related Predictors for RTW

Uni- and multivariable logistic regression analyses are shown in Table A1 (Appendix A) and Table 5, respectively. In univariable analysis, RTW was lower for participants living in less central areas compared to those living centrally in the total population (OR 0.37, 95% CI 0.2–0.69) and in both the dominant head injury (OR 0.41, 95% CI 0.17–0.97) and orthopaedic injuries subgroups (OR, 0.2, 95% CI 0.06–0.69).

However, this association remained significant only within the extremity and spine subgroup (OR 0.2, 95% CI 0.04–0.9) when adjusting for possible confounding factors.

Investigating prognostic factors for RTW in multivariable logistic regression showed that age was a significant predictor of RTW in the total study population and the head injury subgroup. Having a blue-collar job was a significant negative prognostic factor both in the total population and all injury subgroups in uni- and multivariable analysis. Of injury-related variables, the total number of injuries and RCS-E Trauma baseline were significant in multivariable analysis for the total population.

### 3.4. Function and RTW

Function at 12 months was lower for participants living less centrally compared to those living more centrally (mean WHODAS 11-item sum score 5 vs. 3, *p* = 0.023) (Table 4).

**Figure 2:**
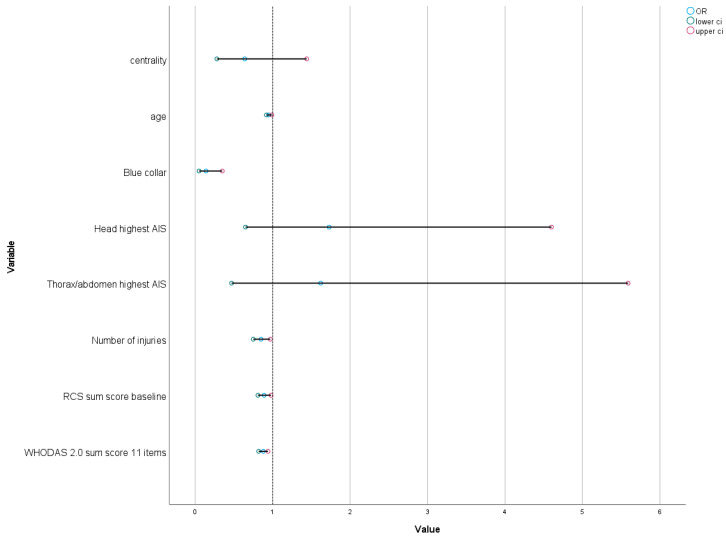
Predictors for return to work at 12 months in multivariate analysis. N = 243.

When added to the multivariable model for the total population, lower function (higher WHODAS 11-item sum scores) was significantly negatively associated with RTW (OR 0.88, 95% CI 0.82–0.94, *p* < 0.001) (Figure 2). The model fit and explanation of variance within the model were good with Hosmer and Lemeshow test and Nagelkerke R^2^ results of 0.614 and 0.483, respectively.

## 4. Discussion

This study examined RTW in patients treated for moderate to severe traumatic injuries at two Norwegian trauma centres. We investigated centrality, demographics and injury-related variables as predictors for RTW. After 6 months, 68% of participants had returned to work. RTW at the 12-month follow-up was 77%. Univariable analysis revealed that participants living in less central areas were less likely to return to work than those living in central areas. However, multivariate analysis showed that demographic factors (length of education and type of job) and injury-related variables (NISS, total number of injuries and rehabilitation complexity) were the main contributors to the differences in RTW.

### 4.1. Return to Work or School at 6 and 12 Months after Traumatic Injury

The RTW rate in this study (77%) is consistent with previous results in the literature. De Munter et al. [5] included patients with less severe injuries (median ISS 5) and found an RTW rate of 76% after 12 months. In a study by Collie et al. [6] patients with a median ISS of 17 RTW was 60% after 6 months and 66% after twelve months. Holtslag et al. [7] found that 80% of patients with severe injuries (ISS > 16) returned to work after approximately 15 months (22% part-time). Soberg et al. [11] also found a 74% RTW rate, with 28% returning to full-time work after one year in patients with severe injuries (NISS > 15). Despite differences in the populations investigated, our results are in line with these studies indicating an RTW rate of 65% to 80% after one year. However, the higher proportion of negative risk factors in patients not participating in the 12-month follow-up may lead to an overestimation of the RTW rate in the present study. Furthermore, the increase in RTW between 6 and 12 months indicates the need for long-term follow-up of patients with negative risk factors to ensure their successful reintegration into work.

### 4.2. Centrality as an Early Predictor for RTW at 12 Months Post-Injury

In this study, participants living in less central areas had lower RTW rates than those living centrally. However, this difference was not significant in multivariable analysis. In contrast, studies based on insurance data have reported differences in RTW between central and rural areas, these have more often reported a benefit to RTW from living in central areas, Refs. [17,18,19] but the opposite has also been found [15]. Demographic and injury-related variables that differed between participants from urban and central areas were significant predictors for RTW and probably explain the observed differences in rates. For instance, rural municipalities in Norway have an older population: the average age is higher in the least central municipalities, compared with the most central [39]. People living in rural areas also have lower average education levels [40] and are more likely to work blue-collar jobs.

### 4.3. Predictive Impact of Demographic and Early Injury-Related Co-Variables and Association of Function with RTW

In the multivariable analysis for the total study population, both age and type of job were significant demographic variables, while the total number of injuries and rehabilitation complexity at baseline were significant injury-related variables. In other studies with comparable patient populations, higher age [10,11,41], female gender [5,10], lower level of education [5,11], physically demanding job [6,10], pre-injury comorbidity [5,6,10,41] and higher injury severity [5,41] have been previously shown to have a negative predictive effect.

In this study, participants living in less central areas had more severe injuries. This may be because patients with moderate injuries are treated in local hospitals and not transferred to the central regional trauma centres, for participants living in central areas, the trauma centre may be their local hospital, and they may be treated there even with less severe injuries.

We estimated acute rehabilitation needs in this population using the RCS-E Trauma and found that higher needs were associated with lower RTW. There are no similar studies with which to compare these results. We therefore concluded that the RCS-E Trauma, as used here, is an indicator of injury severity.

In this study, functioning was significantly associated with RTW, even when controlling for demographic and injury-related early predictors. In contrast, Holtslag et al. [7] did not find the same association in a population of severely injured patients after approximately 15 months. Other studies, however, have reported that low functional level/disability is a strong negative predictor of RTW [22].

### 4.4. Strengths and Limitations

The prospective population-based study design, including two of four Norwegian trauma centres, and a high participation rate of 83% are strengths of this study. The small sample size, however, especially in the injury-type subgroups, limits the analytical depth and predictor analysis. There may also be a selection bias, as non-responders differed significantly in several demographic pre-injury variables. Non-responders more often had lower education and severe pre-morbidity, which decreases the probability of returning to work; they were however also younger, which has the opposite effect. There were no significant differences in centrality and injury-related variables. Overall, this may have led to lower RTW in the non-responder group.

## 5. Conclusions

The proportion of participants who had returned to work after one year was high in all major trauma groups. Demographic and injury-related factors were more important predictors for RTW than the centrality of residence. Blue-collar workers and patients with multiple injuries and high rehabilitation complexity should be given special attention in order to promote their vocational rehabilitation, job retention and return-to-work programmes ensuring the right of persons with disabilities to work. More research is needed to develop evidence-based RTW programmes customised to the vocational for the general trauma population in Norway.

## Figures and Tables

**Table 1:** Demographic characteristics of working or studying patients aged 18 to 70 with moderate to severe trauma, who participated in the 12-month follow-up.

N = 243	
Age, Years	
Median (range)	47 (18–69)
Mean (SD)	45 (14)
Gender, n (%)	
Female	51 (21)
Male	192 (79)
Education, n (%)	
Lower education ≤ 13 years	109 (45)
High school/university > 13 years	133 (55)
Unknown	1 (0.4)
Marital status, n (%)	
Living with someone	165 (68)
Living alone	78 (32)
Type of occupation at time of injury n (%)	
Working	220 (91)
Studying	23 (9)
Type of job n (%)	
Blue collar	102 (42)
White collar	140 (58)
Missing	1 (0.4)
Centrality index score (NCI) n(%)	
1 (most central)	106 (44)
2	43 (18)
3	62 (26)
4	15 (6)
5	13 (5)
6 (most rural)	4 (2)
RTW, n (%)	
No RTW	56 (23)
- of which receiving disability pension	12 (5)
- of which retired	11 (5)
- of which on sick leave	33 (14)
Yes RTW	187 (77)
- of which back in full work	151 (62)
- of which partly back in work	33 (14)

**Table 2:** Injury-related characteristics of working or studying patients who participated in the 12-month follow-up.

N = 243	
Substance Use at time of the Accident, n (%)	49 (20)
Pre-injury ASA, n (%)	
ASA I–II, no disability	236 (97)
ASA III–V, disability	7 (3)
New Injury Severity Score (NISS)	
Mean	25 (13)
Median (range)	22 (10–75)
Moderate NISS 10–15, n (%)	58 (24)
Severe NISS ≥ 16, n (%)	185 (76)
Injury Severity Score (ISS)	
Median (range)	14 (4–59)
ISS ≥ 16, n (%)	118 (48)
Number of injuries median (range)	5 (1–23)
AIS organ area with highest score, n (%)	
Head	103 (42)
Extremities/spine without spinal cord injury	61 (25)
Spinal cord	15 (6)
Face	4 (2)
Thorax/abdomen	60 (25)
WHODAS 12-items at 12 months, median (IQR)	2 (0–6)
WHODAS 11-items at 12 months, median (IQR)	1 (0–5)

ASA = American Society of Anaesthesiologists Physical Status Classification System. AIS = Abbreviated Injury Score. ISS = Injury Severity Score. NISS = New Injury Severity Score. WHODAS = World Health Organisation Disability Assessment Schedule 2.0.

**Table 3:** Return-to-work 6- and 12-months post-injury for dominating injury types in 223 adults in work/education at the time of the injury and who attended both follow-ups.

	RTW 6 Months n (Row%)			RTW 12 Months n (Row%)		
Dominating Type of Injury	No	Partly	Full	No	Partly	Full
Total N = 223	71 (32)	60 (27)	92 (41)	48 (22)	32 (14)	140 (63)
Head n = 97	40 (41)	27 (28)	30 (31)	27 (28)	19 (20)	51 (53)
Extremities and spine without spinal cord n = 54	17 (32)	14 (26)	23 (43)	11 (20)	9 (17)	33 (61)
Spinal cord n = 14	3 (21)	7 (50)	4 (29)	2 (14)	2 (14)	10 (71)
Face n = 3 *	1	0	2	0	0	3
Thorax/abdomen n = 55	9 (16)	12 (22)	33 (60)	6 (11)	6 (11)	43 (78)

* percentages not reported because of the low total number.

**Table 4:** RTW after 12 months and demographic and injury-related variables in relation to centrality index for 243 participants who attended the 12-month follow-up.

	Centrality Index 1–2 N = 149	Centrality Index 3–6 N = 94	*p*
RTW yes (%)	125 (84)	62 (66)	**0.001**
Age in years, mean (SD)	46 (14)	44 (15)	0.512
Female Gender, n (%)	34 (23)	17 (18)	0.377
Living alone, n (%)	54 (36)	24 (26)	0.082
Education ≤ 13 years, n (%)	50 (34)	59 (63)	**<0.001**
Blue-collar jobb, n (%)	47 (32)	55 (59)	**<0.001**
ASA III-IV, n (%)	4 (3)	3 (3)	0.818
Substance use at the time of the injury, n (%)	28 (19)	21 (22)	0.502
NISS, mean (SD)	23 (12)	28 (14)	**0.002**
Number of injuries totally, mean (SD)	6 (3)	7 (3)	**0.002**
Head, n (%)	59 (40)	44 (47)	0.268
Extremities/spine without spinal cord, n (%)	38 (26)	23 (25)	0.856
Spinal cord, n (%)	7 (5)	8 (9)	0.229
Face, n (%)	3 (2)	1 (1)	1.0 *
Thorax/abdomen, n (%)	42 (28)	18 (19)	0.112
Estimated RCSE Trauma Baseline, mean (SD)	7 (6)	10 (5)	**<0.001**
WHODAS 11-item 12 months, mean (SD)	3 (7)	5 (6)	**0.023**

*p*-values in bold are significant; * Fisher’s exact test (2-sided).

**Table 5:** Return to work rates at 12 months related to centrality in 243 participants, controlled for selected early predictors in multivariable logistic regression analysis.

	Total			Head			Extremities/Spine without Spinal Cord		
	N = 243			N = 103			n= 61		
	RTW Yes/No (%/%) 187/56 (77/23)			RTW Yes/No (%/%) 73/30 (71/29)			RTW Yes/No (%/%) 46/15 (75/25)		
	OR	95% CI	*p*-Value	OR	95% CI	*p*-Value	OR	95% CI	*p*-Value
Centrality index (NCI)									
1 and 2 (ref.)/3–6	0.71	0.34–1.50	0.370	0.56	0.19–1.6	0.276	0.2	0.04–0.9	**0.036**
Age mean	0.95	0.92–0.98	**<0.001**	0.93	0.89–0.97	**0.001**			
Education low/high n = 242	0.58	0.24–1.41	0.232						
Blue collar/hvite collar n = 242	0.23	0.09–0.57	**0.002**	0.29	0.1–0.85	**0.025**	0.12	0.02–0.56	**0.007**
NISS mean	0.99	0.95–1.03	0.487	0.92	0.89–0.96	**<0.001**			
Number of injuries totally mean	0.85	0.75–0.96	**0.010**				0.74	0.56–0.97	**0.030**
Highest AIS head mean	2.12	0.8–5.62	0.133						
Highest AIS Thorax/abdomen mean	2.38	0.75–7.6	0.144						
Sum RCS baseline median	0.87	0.79–0.95	**0.003**						

Model fit: Hosmer Lemeshow 0.263, H. and L. 0.175, Hosmer Lemeshow 0.916. Degree of pseudo-explained variance, Nagelkerke R^2^ 0.416, Nagelkerke R^2^ 0.429, Nagelkerke R^2^ 0.454.

## Data Availability

The datasets generated and/or analysed in the current study are not publicly available due to the sensitivity of the material.

## Appendix A

**Table A1:** Results from univariable logistic regression investigating possible prognostic factors with return to work at 12 months in the total population (n = 243) and within subgroups of injuries.

	Total Population				Head (n = 103)				Extremities and Spine without Spinal Cord, (n = 61)				Thorax/Abdomen, (n = 60)			
	RTW				RTW				RTW				RTW			
Factors	Yes (n = 187)	No (n = 56)	OR (95% CI)	*p*-Value	Yes (n = 72)	No (n = 31)	OR (95% CI)	*p*-Value	Yes (n = 46)	No (n = 15)	OR (95% CI)	*p*-Value	Yes (n = 53)	No (n = 7)	OR (95% CI)	*p*-Value
Centrality, n (%)																
1–2	125 (66.8)	24 (42.9)	1.0 (ref)		46 (64)	13 (42)	1.0 (ref)		33 (72)	5 (33)	1.0 (ref)		36 (68)	6 (86)	1.0 (ref.)	
3–6	62 (33.2)	32 (57.1)	0.37 (0.2–0.69)	**0.002**	26 (36)	18 (58)	0.41 (0.17–0.97)	**0.041**	13 (28)	10 (67)	0.2 (0.06–0.69)	**0.011**	17 (32)	1 (14)	2.83 (0.32–25.42)	0.352
Age in years, mean (SD)	44 (14)	50 (13)	0.97 (0.95–0.99)	**0.006**	43 (15)	53 (13)	0.95 (0.92–0.98)	**0.003**	40(15)	44 (14)	0.99 (0.95–1.03)	0.462	47 (12)	51 (10)	0.97 (0.9–1.05)	0.406
Gender n (%)																
Female	39 (21)	12 (21)	0.97 (0.47–2.0)	0.926	15 (21)	6 (19)	1.1 (0.38–3.16)	0.864	10 (22)	3 (20)	1.11 (0.26–4.72)	0.886	12 (23)	3 (43)	0.39 (0.14–4.41)	0.258
Male	148 (79)	44 (79)	1.0 (ref.)		57 (79)	25 (81)	1.0 (ref.)		36 (78)	12 (80)	1.0 (ref.)		41 (77)	4 (57)	1.0 (ref.)	
Living alone n (%)																
Yes	61 (33)	17 (30)	0.9 (0.47–1.72)	0.75	23	6	0.9 (0.47–1.72)	0.75	18 (39)	5 (33)	0.78 (0.23–2.65)	0.688	18 (34)	2 (29)	0.78 (0.14–4.41)	0.777
No	126 (67)	39 (70)	1.0 (ref.)		49	22	1 (ref.)		28 (61)	10 (67)	1.0 (ref.)		35 (66)	5 (71)	1.0 (ref.)	
Education n (%)																
Low	72 (39)	37 (66)	0.32 (0.17–0.61)	**<0.001**	28 (39)	19 (61)	0.4 (0.17–0.95)	**0.039**	21 (46)	12 (80)	0.21 (0.05–0.85)	**0.028**	17 (33)	4 (57)	0.36 (0.7–1.81)	0.218
High	114 (61)	19 (34)	1.0 (ref.)		44 (61)	12 (39)			25 (54)	3 (20)	1.0 (ref.)		35 (67)	3 43)	1.0 (ref.)	
Type of job n (%)																
Blue collar	63 (34)	39 (70)	0.22 (0.12–0.43)	**<0.001**	28 (39)	20 (65)	0.35 (0.15–0.84)	**0.19**	13 (28)	12 (80)	0.098 (0.02–0.41)	**0.001**	15 (29)	5 (71)	0.16 (0.3–0.93)	**0.041**
White collar	123 (66)	17 (30)	1.0 (ref.)		44 (61)	9 (35)	1.0 (ref.)		33 (72)	3 (20)	1.0 (ref.)		37 (71)	2 (29)	1.0 (ref.)	
ASA, n (%)																
III–IV	5 (3)	2 (4)	0.74 (0.14–3.91)	0.721	1 (1)	2 (6)	0.2 (0.18–2.34)	0.202	Not analysable with logistic regression				Not analysable with logistic regression			
I–II	182 (97)	44 (96)	1.0 (ref.)		71 (99)	29 (94)	1.0 (ref.)									
Substance use at time of accident n (%)																
yes	36 (19)	13 (23)	0.78 (0.38–1.61)	0.506	25 (35)	12 (39)	0.84 (0.35–2.01)	0.699	Not analysable with logistic regression				4 (8)	1 (14)	0.49 (0.05–5.13)	0.552
no	151 (81)	43 (77)	1.0 (ref.)		47 (65)	19 (61)	1.0 (ref.)						49 (92)	6 (86)	1.0 (ref.)	
NISS mean (SD)	23 (11)	31 (16)	0.95 (0.93–0.97)	**<0.001**	27 (12)	40 (15)	0.94 (0.91–0.97)	**<0.001**	17 (8)	18 (87)	0.99 (0.92–1.06)	0.782	22 (8)	22 (4)	1.0 (0.9–1.13)	0.875
Number of injuries totally mean (SD)	5 (3)	8 (4)	0.82 (0.74–0.9)	**<0.001**	6 (3)	8 (4)	0.87 (0.78–0.98)	**0.019**	5 (3)	7 (2)	0.76 (0.61–0.94)	**0.014**	5 (2)	8 (3)	0.69 (0.49–0.96)	**0.03**
Sum RCS baseline median (IQR)	7 (1–11)	12 (9–15)	0.84 (0.78–0.89)	**<0.001**	10 (1–14)	13 (10–16)	0.86 (0.79–0.95)	**0.002**	8 (1–10)	10 (1–109	0.9 (0.79–1.03)	0.111	1 (1–7)	11 (8–15)	0.49 (0.28–0.85)	**0.011**
Highest AIS																
Head	72	31	0.55 (0.3.1.0)	0.052												
Extremities/spine	46	25	0.89 (0.45–1.75)	0.725												
Thorax/abdomen	53	7	2.77 (1.18–6.5)	**0.019**												
Function																
WHODAS 2.0 sum mean (SD)	2.5 (4.4)	8.9 (9.4)	0.86 (0.81–0.92)	**<0.001**	1.7 (2.2)	10.4 (10.6)	0.71 (0.59–0.84)	**<0.001**	4.4 (7.4)	7.6 (7.5)	0.42 (0.23–0.78)	**0.006**	1.8 (2.6)	5.6 (8.8)	0.5 (0.22–1.113)	0.096

Significant *p*-values are written in bold.
